# Dysregulation of IFN System Can Lead to Poor Response to Pegylated Interferon and Ribavirin Therapy in Chronic Hepatitis C

**DOI:** 10.1371/journal.pone.0019799

**Published:** 2011-05-13

**Authors:** Koji Onomoto, Shiho Morimoto, Takahisa Kawaguchi, Hidenori Toyoda, Masami Tanaka, Masahiko Kuroda, Kazuko Uno, Takashi Kumada, Fumihiko Matsuda, Kunitada Shimotohno, Takashi Fujita, Yoshiki Murakami

**Affiliations:** 1 Institute for Viral Research and Graduate School of Bioscience, Kyoto University, Kyoto, Japan; 2 Center for Genomic Medicine, Kyoto University Graduate School of Medicine, Kyoto, Japan; 3 Department of Gastroenterology, Ogaki Municipal Hospital, Ogaki, Japan; 4 Department of Molecular Pathology, Tokyo Medical University, Tokyo, Japan; 5 Louis Pasteur Center for Medical Research, Kyoto, Kyoto, Japan; 6 Research Institute, Chiba Institute for Technology, Narashino, Japan; 7 Research Institute for Science and Engineering, Waseda University, Tokyo, Japan; Massachusetts General Hospital, United States of America

## Abstract

**Background:**

Despite being expensive, the standard combination of pegylated interferon (Peg-IFN)- α and ribavirin used to treat chronic hepatitis C (CH) results in a moderate clearance rate and a plethora of side effects. This makes it necessary to predict patient outcome so as to improve the accuracy of treatment. Although the antiviral mechanism of genetically altered IL28B is unknown, IL28B polymorphism is considered a good predictor of IFN combination treatment outcome.

**Methodology:**

Using microarray, we quantified the expression profile of 237 IFN related genes in 87 CH liver biopsy specimens to clarify the relationship between IFN pathway and viral elimination, and to predict patients' clinical outcome. In 72 out of 87 patients we also analyzed IL28B polymorphism (rs8099917).

**Principal Findings:**

Five IFN related-genes (IFI27, IFI 44, ISG15, MX1, and OAS1) had expression levels significantly higher in nonresponders (NR) than in normal liver (NL) and sustained virological responders (SVR); this high expression was also frequently seen in cases with the minor (TG or GG) IL28B genotype. The expression pattern of 31 IFN related-genes also differed significantly between NR and NL. We predicted drug response in NR with 86.1% accuracy by diagonal linear discriminant analysis (DLDA).

**Conclusion:**

IFN system dysregulation before treatment was associated with poor IFN therapy response. Determining IFN related-gene expression pattern based on patients' response to combination therapy, allowed us to predict drug response with high accuracy. This method can be applied to establishing novel antiviral therapies and strategies for patients using a more individual approach.

## Introduction

Hepatitis C virus (HCV) infection affects more than 3% of the world population. Without suitable treatment, chronic hepatitis C (CH) frequently leads to the development of chronic liver diseases such as liver cirrhosis (LC) and hepatocellular carcinoma (HCC) [1]. The current standard treatment for CH is a combination of pegylated-IFN (Peg-IFN)- α and ribavirin (hereafter CH combination therapy). Over a 15-year observation period, the rate of hepatocarcinogenesis was found to be significantly lower in sustained viral responders (SVR) and relapse (R) patients than in non responders (NR) and interferon (IFN) untreated patients [2]. However, CH combination therapy achieves a sustained virological response in 50–55% of patients with HCV genotype 1b infection [3]. Consequently, this creates a pressing need to develop alternative strategies for treating CH.

IFN Type-I and III play various important immunomodulatory roles in both innate immune and acquired immune responses. Four main effector pathways of the IFN-mediated antiviral response have been recognized by gene targeting studies: the Mx GTPase pathway, the 2′, 5′-oligoadenylate-synthetase-directed ribonuclease L (OASL) pathway, the protein kinase R (PKR) pathway and the interferon stimulated gene (ISG) 15 ubiquitin-like pathway. These effector-pathways individually block viral transcription, degrade viral RNA, inhibit translation and modify protein function to control all steps of viral replication [4]–[5].

IFN treatment for CH usually results in a high incidence of side effects; therefore, it is important to adjust IFN treatment accurately using a prediction method. Viral factors (HCV genotype, pretreatment viral load, and sequence of HCV gene core and NS5A), [6]–[7] host factors (obesity, cirrhosis, ethnic background, serum cytokine levels, liver fibrosis grades) [8], and treatment factors (adequate course of treatment, adherence to the treatment, management of side effects) [9] has been utilized in prior research to predict the outcome of combination therapy. Hepatic microRNA expression pattern before anti-viral treatment has also been utilized as a prediction biomarker of drug response in CH [10], while other studies have shown that there is a possible association between two SNPs near the gene interleukin 28B (IL28B) on chromosome 19 and lack of response to combination therapy [11]–[13].

In this study, we evaluated the IFN related gene expression profiles in CH patients before administering CH combination treatment. After the anti-viral therapy, patients were classified according to their clinical outcome: sustained viral response (SVR), relapse (R), and non responder (NR). It was observed that in the NR group, the expression level of some IFN related genes was significantly higher than that in normal liver (NL) groups, and that the expression level of the other IFN related genes was significantly lower than in NL. Moreover, the significantly high expression of IFN related genes was associated with low response to combination therapy. This suggests that dysregulation of the IFN system can be related to cases of CH combination therapy failure.

## Results

In order to provide specific information with less data analysis, we developed a custom-made focused DNA microarray called Genopal (Mitsubishi Rayon, Tokyo, Japan) using genes that target human innate-immunity. Based on the results from the expression profiles, we carefully selected 237 gene probes (materials and methods) by activating RIG-I with Agilent DNA microarray. A microarray platform was used to establish IFN-related gene expression profiles in the specimens collected from the 87 CH and 5 NL samples (Table 1). The results of the analysis of these genes using the DNA chip strongly correlated with those obtained by real-time PCR (Pearson's correlation coefficient R2 = 0.996, P<0.0001; data not shown).

**Table 1:** Clinical characteristics of patients.

Characteristics	SVR (n = 38)	R (n = 26)	NR (n = 23)	NL (n = 5)
Age	56.7±10.3	61.3±8.6	60.8±7.8	57.2±9.5
Male (%)	28 (61%)	11 (39%)	9 (36%)	3(60%)
Weight (kg)	59.5±8.9	57.2±10.3	55.7±7.2	ND
HCV RNA (x10^6^ copies/ml)	2.00±2.07	1.79±1.02	1.55±0.95	ND
Fibrosis stage				
F 0	1	1	1	
F 1	29	13	10	
F 2	9	7	5	
F 3	6	4	6	
F 4	0	0	1	
WBC(×10^3^/mm^3^)	5.42±1.63	5.23±1.25	4.69±1.13	ND
Hemoglobin (g/dl)	14.3±1.14	13.5±1.35	13.6±1.09	ND
Platelet (×10^4^/mm^3^)	16.7±5.3	16.6±4.0	15.0±5.7	ND
AST (IU/L)	59.2±51.0	48.7±30.1	57.4±29.7	ND
ALT (IU/L)	80.8±93.7	49.3±29.6	69.1±44.4	ND
γGTP (IU/L)	60.3±74.2	41.2±29.7	76.2±60.2	ND
ALP (IU/L)	255±74.0	246±71.3	314±144	ND
Total bilirubin (mg/dl)	0.66±0.22	0.73±0.31	0.69±0.19	ND
Albumin (g/dl)	4.20±0.34	4.14±0.25	4.02±0.48	ND

Abbreviations; NR, non-virological responder; R, relapse; SVR, sustained virological responder; AST, aspartate aminotransferase;ALT, alanine aminotransferase; WBC, white blood cell; ALP, alkaline phosphatase; γGTP, gamma-glutamyl transpeptidase; ND, not detected.

### IFN related genes associated with the final response to combination therapy

**Figure 1:**
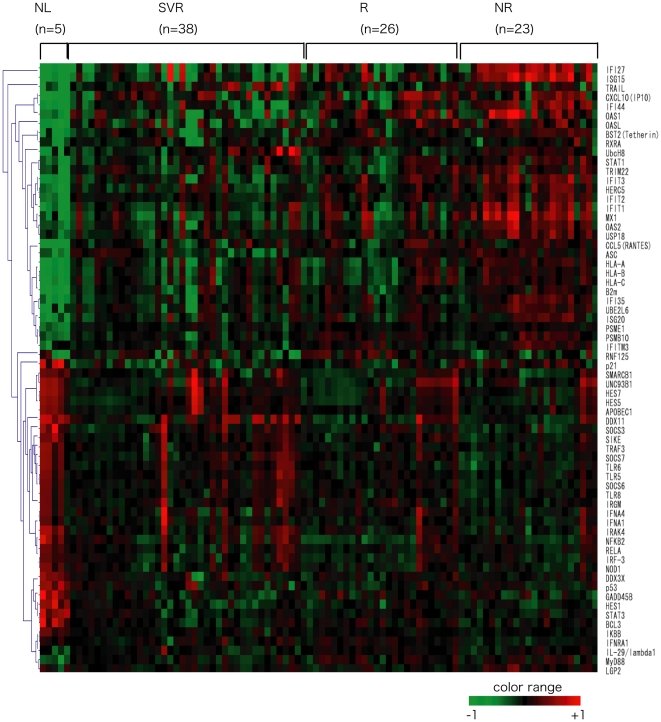
Clustering of IFN related gene expression. Clustering of CH patients according to the expression profiles of the 66 genes that showed significant differences among SVR, R, NR, and NL. Vertical bars represent the IFN related genes and the horizontal bars represent the samples. Green bars reflect down-regulated genes and red bars up-regulated genes.

We determined unique IFN gene expression patterns for liver specimens with or without HCV based on the final virological response to the combination therapy. The expression level of 66 genes significantly differed among NR, R, SVR, and normal liver (NL) groups (Figure 1). To clearly identify the IFN-related genes associated with the clinical outcome, we extracted genes that showed significant differences (p<0.05). It was observed that the expression level of 5 genes (myxovirus (influenza virus) resistance 1 (MX1), 2″,5″-oligoadenylate synthetase 1 (OAS1), ISG15 ubiquitin-like modifier (ISG15), interferon, alpha-inducible protein 27 (IFI27), and interferon, alpha-inducible protein 44 (IFI44)) were significantly higher in NR than in SVR samples (Table 2). The expression levels of 3 genes (MX1, IFI27, and ISG15) were significantly higher in NR than in R samples (Table 2). We also analyzed the IFN-related genes expression pattern according to the grade of inflammation or stage of fibrosis, however, no significant differences was observed between the two (data not shown).

**Table 2:** Extracted genes related to the clinical outcome with a fold change greater than or equal to 1.5 between two groups (NR/SVR, NR/R) (p<0.05).

Accession No.	gene	symbol	fold change (NR/SVR)	p-value
NM_006417.4	interferon, alpha-inducible protein 44	IFI44	2.13	2.01E-03
NM_005532.3	interferon, alpha-inducible protein 27	IFI27*	2.37	2.01E-03
NM_016816.2	2″,5″-oligoadenylate synthetase 1, 40/46kDa, transcript variant 1	OAS1	2.51	1.36E-02
NM_005101.2	ISG15 ubiquitin-like modifier	ISG15*	2.68	1.18E-03
NM_002462.2	myxovirus (influenza virus) resistance 1, interferon-inducible protein p78 (mouse)	MX1*	2.71	1.57E-03
Accession No.	gene	symbol	fold change (NR/R)	p-value
NM_002462.2	myxovirus (influenza virus) resistance 1, interferon-inducible protein p78 (mouse)	MX1*	2.27	1.11E-03
NM_005532.3	interferon, alpha-inducible protein 27	IFI27*	2.33	1.69E-03
NM_005101.2	ISG15 ubiquitin-like modifier	ISG15*	2.5	1.11E-03

Asterisk deposits extracted genes that are common to both SVR and NR and to NR and R.

### Comparison of IFN related genes between CH and NL

We also compared the gene expression pattern in NR and NL. After extracting genes with a fold change <1/3, 3< and p-value<0.05, we found that the expression level of 6 genes (growth arrest and DNA-damage-inducible, beta (GADD45B), hairy and enhancer of split 1 (HES1), B-cell CLL/lymphoma 3 (BCL3), signal transducer and activator of transcription 3 (STAT3), suppressor of cytokine signaling 3 (SOCS3), and DEAD/H (Asp-Glu-Ala-Asp/His) box polypeptide 11 (DDX11)) was significantly lower in NR than in NL. The expression level of SOCS3 and DDX11 in NR was significantly lower than in SVR. The expression level of 25 genes were significantly higher in NR than in NL. The expression levels of most of these genes were significantly higher in NR than in SVR, but the expression level of tumor necrosis factor (ligand) superfamily, member 10 (TRAIL), major histocompatibility complex, class I, C (HLA–C), major histocompatibility complex, class I, B (HLA–B), and chemokine (C-X-C motif) ligand 10 (CXCL10 (IP10)) were similar in NR and SVR samples (Table 3).

**Table 3:** List of genes that had significantly different expression levels in NR and NL (fold change <1/3, 3<, and p<0.05).

symbol	NR/NL(fold change)	NR/NL(t-test)	NR/SVR(fold change)	NR/SVR(t-test)
GADD45B	0.20	1.14E-02	1.01	NS
HES1	0.26	1.26E-03	0.97	NS
BCL3	0.26	1.84E-02	1.02	NS
STAT3	0.26	5.81E-04	0.97	NS
SOCS3	0.27	7.96E-03	0.68	2.15E-02
DDX11	0.28	4.33E-05	0.59	9.52E-03
TRIM22	3.06	2.91E-03	1.37	7.97E-03
ASC	3.19	1.35E-03	1.33	4.07E-03
UBE2L6	3.32	1.06E-02	1.41	1.01E-03
STAT1	3.38	6.04E-04	1.33	1.86E-02
ISG20	3.64	2.42E-04	1.42	2.37E-03
TRAIL	3.81	2.08E-02	0.78	NS
OAS2	4.02	2.91E-03	1.89	1.07E-04
IFIT2	4.60	1.48E-03	1.56	8.34E-05
BST2(Tetherin)	5.14	8.17E-03	1.49	5.67E-04
IFI35	5.29	1.35E-03	1.63	2.37E-05
HERC5	5.32	1.16E-03	1.68	4.07E-05
MX1	6.21	1.33E-03	2.94	8.46E-07
HLA-C	6.49	6.34E-04	1.21	NS
CCL5(RANTES)	6.73	5.48E-04	1.25	3.77E-02
HLA-B	6.84	4.91E-04	1.22	NS
OAS1	7.80	5.52E-04	2.75	1.92E-04
HLA-A	8.49	5.92E-05	1.41	9.08E-04
B2m	9.09	7.78E-04	1.25	1.89E-02
IFIT1	9.42	1.86E-03	2.11	1.41E-05
OASL	10.38	3.97E-06	1.48	1.24E-02
IFIT3	10.45	4.33E-05	2.11	5.63E-06
CXCL10(IP10)	15.67	8.89E-07	1.28	NS
IFI44	17.00	9.40E-05	2.22	4.83E-06
ISG15	21.12	1.05E-04	2.85	3.99E-05
IFI27	43.74	1.80E-05	2.56	5.62E-05

### Validation of the microarray result by real-time qPCR

The five genes (ISG15, MX1, OAS1, IFI27 and IFI44) with the largest difference in fold change between NR and SVR groups were chosen to confirm the microarray results using real-time qPCR. The result from real-time qPCR supported the results from the microarray analysis (Figure S1).

### Prediction of the clinical outcome by DLDA

We attempted to simulate the clinical outcome of the CH combination therapy using diagonal linear discriminant analysis (DLDA). Patients were randomly divided into TS (training set) and VS (validation set) (Table 4) in the order in which their samples were obtained. Samples within each group were then classified as NR or non-NR (SVR+R). DLDA showed that the accuracy, sensitivity, specificity, positive and negative predictive value of these two classifications were 86.1%, 87.5%, 81.8%, 93.3%, and 69.2% respectively (Table 5). Additionally, we attempted to predict (1) SVR and nonSVR (R+NR), and (2) SVR, R, and NR by DLDA. The accuracy with which patients were classified as SVR and nonSVR, was 56.8% and as SVR, R, and NR was 56.9%.

**Table 4:** Characteristics of the training and validation set.

	non NR (SVR+R) group	non NR (SVR+R) group		NR group	NR group	
	average (training set)	average (validation set)	p-value	average (training set)	average (validation set)	p-value
No.	32	32		12	11	
Age	59.3	57.1	0.38	60.6	61.7	0.74
HCVRNA (×10^6^ IU/ml)	1.77	2.08	0.48	1.51	1.52	0.97
AST (IU/L)	44.6	65.3	0.06	55.3	56.9	0.89
ALT (IU/L)	50	87.3	0.05	67.7	66.8	0.96
WBC(×10^3^/mm^3^)	5220	5440	0.57	4610	4860	0.6
Platelet (×10^4^/mm^3^)	15.8	17.6	0.15	15	15.2	0.95
Total bilirubin (mg/dl)	0.71	0.69	0.78	0.68	0.68	0.92
weight	58.1	59.2	0.67	57	53.8	0.28
ALP (IU/L)	251	249	0.92	298	326	0.64
gGTP (IU/L)	48	57.4	0.54	73.3	73.8	0.98
Hemoglobin (g/dl)	13.9	14.1	0.53	13.7	13.5	0.78
Albumin (g/dl)	4.15	4.21	0.41	4.11	3.98	0.52

**Table 5:** Quality of NR-prediction by DLDA.

		Predicted		
		NR	nonNR(SVR+R)	Total
Diagnosed	NR	9	2	11
	nonNR(SVR+R)	4	28	32
	Total	13	30	43

### Genetic variation of IL28B is correlated with the expression of IFN related genes

**Figure 2:**
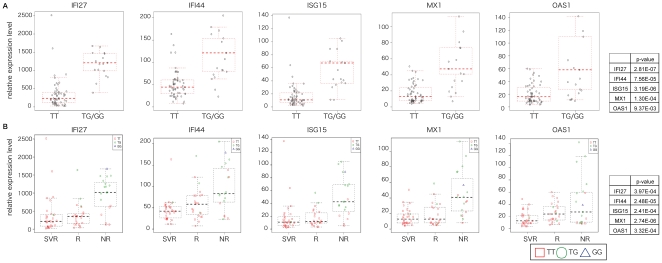
The relationship among the expression of IFN-related genes, IL28B polymorphism and clinical outcome. (A) The relationship between expression of ISG and five related genes (MX1, OAS1, ISG15, IFI27, and IFI44) in the liver of CH patients and IL28B with the major (TT) or minor (TG or GG) genotype (rs8099917) is shown. The p-value of the relationship between gene expression level and IL28B genotype is also depicted. (B) The relationship among the expression level of the above five genes, clinical outcome, and IL28 genotype in individual cases. Red square, green circle, and blue rectangle represent TT, TG, and GG in IL28B genotype, respectively. The p value was calculated from a linear regression employing outcome as an explanatory variable (in which SVR, R and NR are encoded to 0, 1 and 2 respectively) and expression level as the response variable. We tested the null hypothesis that the coefficient of the outcome is 0. Summary table of the p-value is also shown. NS shows no significant difference.

**Figure 3:**
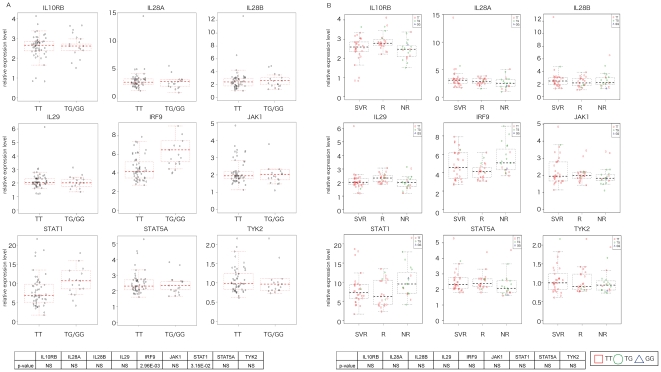
The relationship among the expression of IFN lambda-related genes, IL28B polymorphism and clinical outcome. (A) The relationship between the expression level of IFN lambda related genes (TYK2, STAT5A, STAT1, IL10RB, IL29, IL28A, IL28B, JAK1, and IRF9) in the liver of CH patients and IL28B with genotype. The p-value of the relationship between gene expression level and IL28B genotype is also presented. (B) The relationship among IFN lambda related genes, clinical outcome, and IL28 genotype in individual cases. Summary table of the p-value is also shown. NS was not significantly different.

To examine the relationship between the genetic variation of IL28B and IFN related gene expression, we determined the IL28B polymorphism in 72 patients (Table 6). Patients with the minor genotype of IL28B displayed higher levels of hepatic ISGs expression, whereas patients with the major genotype showed significantly lower expression levels (Figure 2A). In order to further widen our understanding of the above relationship, we significantly identified individual genetic variations in IL28B at the clinical outcome (Figure 2B). We then individually compared the expression level of several IFN-lambda related genes at the clinical outcome with the genetic variation of IL28B. The expression level of interleukin 28A (IL28A), IL28B, interleukin 29 (IL29), interleukin 10 receptor, beta (IL10RB), signal transducer and activator of transcription 1 (STAT1), STAT5A, and tyrosine kinase 2 (TYK2) in IL28B genotype minor allele and major allele did not differ; however, the expression level of STAT5A and IRF9 was significantly higher in IL28B minor allele cases than in major allele (Figure 3A). The expression levels of these nine genes did not significantly differ among the clinical outcomes (NR, R, and SVR) (Figure 3B).

**Table 6:** Result of the IL28B polymorphism (rs8099917).

		rs8099917		
		TT	TG	GG
outcome	NR	7	12	1
	Relapse	18	3	0
	SVR	30	1	0
	Total	55	16	1

**Figure 4:**
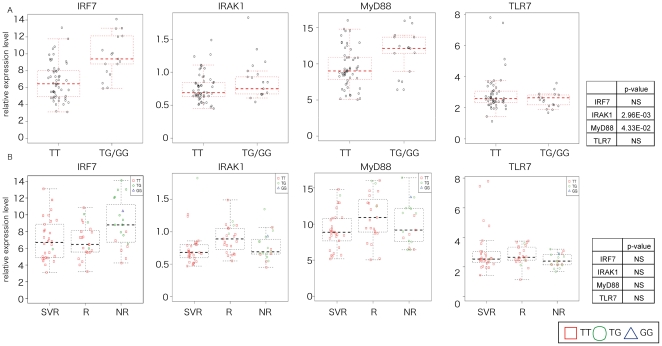
The relationship between the expression level of genes which participate in IFN production (TLR7, MyD88, IRAK1, and IRF7) in the liver of CH patients and IL28B genotype. (A) The relationship between IFN early response genes and clinical outcome is shown. A summary table of the p-value is also presented. NS shows no significant difference. (B) The relationship between IFN early response genes and IL28 genotype is shown. The p-value is also presented.

Finally, in regards to genes which contribute to IFN production (interferon regulatory factor 7 (IRF7), interleukin-1 receptor-associated kinase 1 (IRAK1), myeloid differentiation primary response gene (MyD88), and toll-like receptor 7 (TLR7)) there was not much difference in their expression level prior to CH combination treatment and their expression level at the clinical outcome (Figure 4A) [14]. Unlike IRF7 and MyD88, there was no significant difference in the expression level of IRAK1 and TLR7 according to the IL28B genetic variation (Figure 4B). When we attempted to predict NR and nonNR by using ISG genes with and without IL28B polymorphism using DLDA by using 72 patients (36 patients for training set, 36 patients for validation set). DLDA with IFN related gene and IL28B polymorphism showed that the accuracy, sensitivity, specificity, positive and negative predictive value of these two classifications were 83.3%, 85.1%, 77.8%, 92.0%, 63.6%, respectively (Table 7). DLDA with IFN related gene only showed that the accuracy, sensitivity, specificity, positive and negative predictive value were 83.3%, 81.5%, 88.9%, 95.7%, 61.5%, respectively (Table 8).

**Table 7:** Quality of NR-prediction by DLDA with IFN related gene and IL28B polymorphism A.IFN+IL28B.

		Predicted		
		NR	nonNR	Total
Diagnosed	NR	7	2	9
	nonNR	4	23	27
	Total	11	25	36

**Table 8:** Quality of NR-prediction by DLDA with IFN related gene only.

		Predicted		
		NR	nonNR	Total
Diagnosed	NR	8	1	9
	nonNR	5	22	27
	Total	13	23	36

## Discussion

Our comprehensive analysis identified 66 genes with expression levels that consistently differed depending on the drug response of 87 CH patients and 5 normal liver specimens (Figure 1). Comparing the gene expression pattern in NR and NL showed the expression levels of 31 genes were significantly different (Table 3). In addition, most genes with expression levels in NR that were higher or lower than in NL, also differed between NR and SVR. Therefore, it is possible that innate immunity in the early period of HCV infection strongly influences IFN reaction.

HCV infection induces the impairment of cell subset number and the function of plasmacytoid dendritic cells (PDC) and natural killer cells [15]. The amount of PDC, which are the most potent producers of antiviral Type-I and III IFN [16], decreased in patients' peripheral blood [17], however, PDC was trapped in the HCV infected liver tissue. Therapeutic non-responders had increased PDC migration to inflammatory chemokines before therapy, compared with therapeutic responders [18]. This situation resulted in elevated expressions of IFN-related genes in the CH samples and was associated with their inability to eliminate the virus [19].

Inadequate expression of IFN related genes has been associated with several diseases. High expression of ISG can induce a refractory state in IFN therapy [20] and impaired IFN production leads to high risk of HCV-related hepatocarcinogenesis [21]. Lymphocyte IFN signaling was less responsive in patients with breast cancer, melanoma, and gastrointestinal cancer and these defects may represent a common cancer-associated mechanism of immune dysfunction. Alternately, since immunotherapeutic strategies require functional immune activation, such impaired IFN signaling may hinder therapeutic approaches designed to stimulate anti-tumor immunity [22]. In this way, the dysregulation of the IFN system can influence the progression of diseases and decrease curative effects.

Genes which participate in IFN production (TLR7, MyD88, IRAK1, and IRF7) did not show any significant difference in their expression level prior to CH combination therapy, and their level at the clinical outcome (Figure 4A and 4B). However, the gene expression pattern of down-stream IFN pathway genes (IFI27, IFI44, ISG15, MX1, and OAS1) was significantly different among SVR, R, and NR (Table 2). IFN is usually up-regulated in HCV infected cells; however in some cases, the mechanism that controls IFN becomes abnormal, and the expression levels of IFN and ISG remain high without any curative effect [23]. The ISG family was generally up-regulated in NR compared to SVR [24]–[27] and this high expression of ISG related genes was associated with poor response to IFN therapy in previous, as well as in this present study. ISG15 has been linked to innate immune response to viruses and to cellular response to IFN. Although over-expression of ISG15 enhances the antiviral activity of IFN in vitro in acute infection [28], in chronic infection, extended pre-activation of IFN induced genes leads to dysregulation of the IFN system.

CH therapy is still imperfect at present and therefore suitable prediction methods are necessary to avoid adverse effects. Treatment failure using CH combination therapy is associated with up-regulation of a specific set of IFN-responsive genes thereby making it possible to predict non-response to exogenous therapy [29]. Early gene expression during anti-HCV therapy may elucidate important molecular pathways that might be influencing the probability of achieving a virological response [30]. Our study supports this fact by demonstrating that CH and NL differ fundamentally in their innate response to CH combination therapy.

IFN related gene expression suggests novel aspects of HCV pathogenesis, and form the basis for a subset of genes that can predict treatment response before initiation of combination therapy. After proper external validation, these gene sets may provide the basis for a diagnostic biomarker that can determine early on whether a patient treated with combination therapy is likely to be NR or not. In this respect, what sets our analysis apart is the effect of using DLDA to predict final response with high accuracy in NR and non-NR groups. This prediction showed that the expectation in NR (proportion of actual non-NR versus the predicted number of non-NR) was 93.3% and overall accuracy was 86.1%. In prior report, Dill et al. successfully predicted SVR, but were unable to predict R and NR with high accuracy [31]. In our experiments on the other hand, we predicted NR with high accuracy but were unable to do so for SVR and R. Possible causes for differences between our results and those received by Dill et al. may be (1) the differences in the races of subjects; European patients vs. Japanese patients in our study, (2) the composition of genotype; genotype 1 and 4 vs. genotype 1b in our study, and (3) the difference of the ISG genes extracted.

Genome-wide association studies have described alleic variants near the IL28B gene that are associated with treatment response and with spontaneous clearance of HCV [11]–[13]. In order to clarify the relationship between IL28B polymorphism and drug response, we compared the expression level of IFN-lambda related gene at the clinical outcome with any genetic variation in IL28B. The expression of hepatic ISG and related genes was strongly associated with treatment response and genetic variation of IL28B [32]. Classification of the patients into SVR and NR revealed that ISG expression was conditionally independent of the IL28B genotype. In CH patients in Europe, the expression pattern of genes induced by IFN more accurately predicts CH combination treatment clinical outcome than polymorphism of IL28B [31]. We observed that curative effect prediction using IFN gene expression pattern resulted in high level of accuracy, however, IFN with IL28B or IFN alone resulted in approximately similar levels of accuracy, therefore, the polymorphism of IL28B did not contribute significantly to our prediction. These findings are accordance with Dill et al. results (Table 7). There was an increased expression in NR compared to SVR irrespective of the IL28B genotype. However, there was no significant difference in their expression at the clinical outcome or in the genetic variation of IL28B (Figure 3A and 3B). Genetic variation of IL28B polymorphism is effective in predicting curative effect; however, the reason for this is not fully understood.

In conclusion, comprehensive analysis of IFN related gene showed that dysregulation of the IFN system might be related to treatment failure and that IFN related gene expression before treatment can enable accurate prediction of CH combination therapy clinical outcome. By focusing the full course of treatment on only those patients who have the highest likelihood of achieving SVR, clinicians could potentially reduce the side effects and costs associated with these regimens and provide a more personalized approach to treating CH patients.

## Materials and Methods

### Patients and sample preparation

Eighty seven CH patients with HCV genotype 1b in the Department of Gastroenterology at the Ogaki Municipal Hospital were enrolled between 2004 and 2006 (Table 1). Patients with autoimmune hepatitis, alcohol-induced liver injury, and patients positive for hepatitis B virus associated antigen/antibody or anti-human immunodeficiency virus antibody were excluded. None of the patients had received IFN therapy or immunomodulatory therapy prior to enrollment. Five normal liver specimens were obtained by surgical resection. Three of these were obtained from Osaka City University Hospital and were taken from gall bladder cancer, cholangiocarcinoma, and hemangioma patients whose liver tissue were normal based on histological, virological and blood examination of their liver function. The remaining two normal liver samples were obtained from the Liver Transplantation Unit of Kyoto University Hospital.

Patients' serum HCV RNA was quantified before IFN treatment using Amplicor-HCV Monitor Assay (Roche Molecular Diagnostics Co., Tokyo, Japan). Histological grading and staging of liver biopsy specimens from the CH patients were performed according to the Metavir classification system. Pretreatment blood samples were analyzed to determine the level of aspartate aminotransferase, alanine aminotransferase (ALT), total bilirubin, alkaline phosphatase (ALP), gamma-glutamyl transpeptidase (γGTP), white blood cell (WBC), platelets, and hemoglobin. Written informed consent was obtained from all patients or their guardians and provided to the Ethics Committee of the Graduate School of Kyoto University, Osaka City University and Ogaki Municipal Hospital, who approved this study in accordance with the Helsinki Declaration.

### Treatment protocol

For all enrolled patients, treatment with PegIFN- α-2b (Schering-Plough Corporation, Kenilworth, NJ, USA) and ribavirin (Schering-Plough) was initiated at the beginning of the 1st week and lasted for 48 weeks. PegIFN was administrated at a dose of 1.5 µg kg/week and ribavirin was administrated at the dose recommended by the manufacturer.

### Definition of drug response to therapy

**Figure 5:**
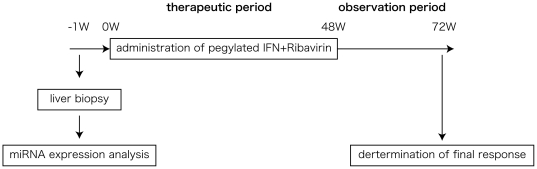
Study design and time line of response to combination therapy. The time frame of liver biopsy, microarray analysis, therapeutic period, observation period after combination therapy, and the judging of clinical outcome is shown.

The patients were classified into the following three groups at the completion of follow-up period (24 weeks): (1) sustained virological responder (SVR): a patient who was negative for serum HCV RNA during the 24 weeks following the completion of the combination therapy; (2) relapse (R): a patient whose serum HCV RNA was negative by the end of the combination therapy but reappeared during the 24 week observation period; and (3) non responder (NR): a patient who was positive for serum HCV RNA during the entire course of the combination therapy (Figure 5). No patients were withdrawn from the study due to side effects or any other reason.

### RNA preparation and real-time qPCR

Total RNA from tissue samples was prepared using a mirVana miRNA extraction Kit (Ambion, Austin, TX, USA) according to the manufacturer's instruction. cDNA was synthesized by Transcriptor High Fidelity cDNA synthesis Kit (Roche, Basel, Switzerland). Total RNA (2 µg) in 11 µl of nuclease free water was added to 1 µl of 50 µM random hexamer and denatured for 10 min at 65°C. The denatured RNA mixture was added to 4 µl of 5x reverse transcriptase buffer, 2 µl of 10 mM dNTP, 0.5 µl of 40 U/ml RNase inhibitor, and 0.5 µl of reverse transcriptase (FastStart Universal SYBR Green Master (Roche) in a total volume of 20 µl. cDNA synthesis was performed for 30 min at 50°C, and enzyme denaturation for 5 min at 85°C. Chromo 4 detector (Bio-Rad, Hercules, CA, USA) was used to detect mRNA expression. Assays were performed in triplicate, and the expression levels of target genes were normalized to that of the ß-actin gene, as quantified using real-time qPCR as internal controls. Nucleotide sequences of primers were as follows: IFI27 (sense) 5′-ctaggccacggaattaaccc-3′, IFI27 (anti-sense) 5′-gactgcagagtagccacaag-3′, IFI44 (sense) 5′-gcatgtaacgcatcaggctt-3′, IFI44 (anti-sense) 5′-ccacaccagcgtttaccaac-3′, ISG15 (sense) 5′-ctttgccagtacaggagctt-3′, ISG15 (anti-sense) 5′-gcccttgttattcctcacca-3′, MX1 (sense) 5′-aatcagcctgctgacattgg-3′, MX1 (anti-sense) 5′-gtgatgagctcgctggtaag-3′, OAS1 (sense) 5′-gtgcgctcagcttcgtactg-3′, OAS1 (anti-sense) 5′-actaggcggatgaggctctt-3′, and β-actin (sense) 5′-ccactggcatcgtgatggac-3′, β-actin (anti-sense) 5′-tcattgccaatggtgatgacct-3′.

### cDNA microarray

RNA was amplified and biotinylated using the MessageAmp-Biotin Enhanced Kit (Ambion). DNA oligonucleotide probes were synthesized onto a DNA microarray chip called Genopal (Mitsubishi Rayon) in order to detect the 237 genes (200 genes on Chip1 and 37 genes on Chip2) related to the innate immune response. Hybridization was carried out overnight at 65°C using Genopal in an hybridization buffer[0.12 M Tris-HCl/0.12 M NaCl/0.05% Tween-20]. After hybridization, Genopal was washed with hybridization buffer twice at 65°C for 20 min followed by washing in 0.12 M Tris-HCl/0.12 M NaCl at 65°C for 10 min. Genopal was then labeled with streptavidin-Cy5 (GE Healthcare Bioscience, Tokyo, Japan). The fluorescent labeled-Genopal was washed for 5 min four times with hybridization buffer at RT and scanned at multiple exposure times ranging from 0 to 40s by DNA microarray reader (Yokogawa Electric Co, Tokyo, Japan). Intensity values with the best exposure condition for each spot were selected. The data presented here have been deposited in NCBI's Gene Expression Omnibus and are accessible through GEO Series accession number GSE20119: http://www.ncbi.nlm.nih.gov/geo/query/acc.cgi?token=xlmbxyyumcwkeba&acc=GSE20119. All data are MIAME compliant, and are also registered with GEO.

### Statistical analysis

To identify the genes that varied significantly among NR, R, SVR and NL groups, one-way ANOVA and Turkey's post hoc tests were used to assess each of the 237 IFN related-genes on the arrays. Benjamini-Hochberg correction for multiple hypotheses testing was applied to all tests. P values <0.05 were considered statistically significant.

### Method of predicting prognosis

The patients were randomly divided into two groups: one was used as a TS and the other VS to calculate the prediction discriminant. A prognosis signature (PS) was defined in terms of the expression levels of the six genes that differed significantly between NR and non-NR groups using post hoc analysis (IFI27, IFI44, interferon-induced protein with tetratricopeptide repeats 3 (IFIT3), ISG15, MX1, OAS1). A prognosis predictor (PP) was computed by applying a diagonal linear DLDA to the TS [33] and then using it to predict the prognoses of the VS. The predicted and actual prognoses of VS patients were compared to obtain the following five measures of prognosis prediction performance: accuracy (proportion of correctly predicted prognoses), sensitivity (proportion of correctly predicted non-NR), specificity (proportion of correctly predicted NR), PPV (proportion of actual non-NR versus predicted non-NR) and NPV (proportion of actual NR versus predicted NR).

### Genetic Variation of IL28B Polymorphism

Genotypes rs8099917 was determined in 72 out of 87 patients by Taqman SNP assays (Applied Biosystems) using a pre-designed and functionally tested probe (ABI assay ID (C_11710096_10). The experiment was carried out according to the manufacturer's instruction.

## Supporting Information

Figure S1 **Real-time qPCR validation of the five IFN related genes.** Each column represents the relative amount of mRNAs normalized to expression level of β-actin. The data shown are means+SD of three independent experiments. Asterisk was indicated to the significant difference at p<0.05.
(TIF)
