# Supplementary figures and images for: Dysregulation of IFN System Can Lead to Poor Response to Pegylated Interferon and Ribavirin Therapy in Chronic Hepatitis C
Source: PLoS One. 2011 May 13;6(5):e19799. doi: 10.1371/journal.pone.0019799 (PMC3094385; doi:10.1371/journal.pone.0019799)

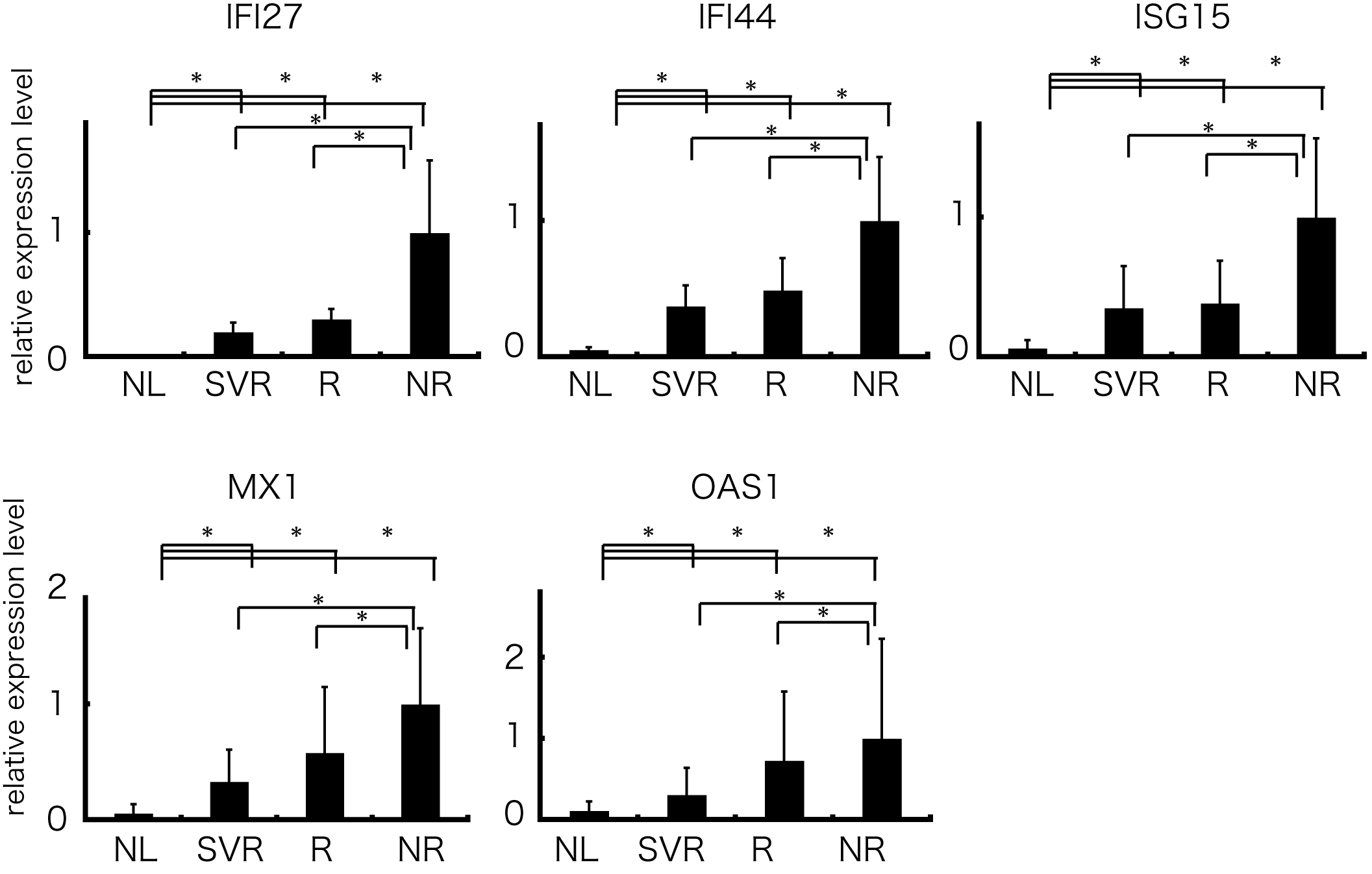

Supplement: Figure S1 — Real-time qPCR validation of the five IFN related genes. Each column represents the relative amount of mRNAs normalized to expression level of β-actin. The data shown are means+SD of three independent experiments. Asterisk was indicated to the significant difference at p<0.05. (TIF) [file pone.0019799.s001.tif]
